# Axenic Cultivation and Pathogenic Assays of *Acanthamoeba* Strains Using Physical Parameters

**Published:** 2013

**Authors:** M Niyyati, H Abedkhojasteh, M Salehi, Sh Farnia, M Rezaeian

**Affiliations:** 1 Department of Medical Parasitology and Mycology, School of Medicine, Shahid Beheshti University of Medical Sciences, Tehran, Iran; 2 Department of Parasitology and Mycology, School of Public Health, Tehran University of Medical Sciences, Tehran, Iran; 3 Center for Research of Endemic Parasites of Iran (CREPI), Tehran University of Medical Sciences, Tehran, Iran

**Keywords:** *Acanthamoeba*, Axenic cultivation, Pathogenicity

## Abstract

**Background:**

The main goal of the present study was to set up an axenic cultivation of *Acanthamoeba* and assess the pathogenic ability of T4 genotypes from different clinical and environmental strains of *Acanthamoeba* using two physical assays.

**Methods:**

Sixteen *Acanthamoeba* isolates including 10 environmental and 6 clinical strains were cultured axenically. Axenic cultivation was performed using Proteosepepton, yeast extract and glucose medium and TY-I-S33culture. Pathogenic survey was done using osmotolerance and thermotolerance assay. Briefly, differentosmolarity (0.5 M and 1 M) of non-nutrient agar plates were performed. One hundred fifty µl of axenic culture were collected and were inoculated in 1% agar medium. For thermotolerance assay 150 µl of amoebas from axenic culture were divided into fresh culture mediums. Cultures were incubated at 37°C and 42 °C. All plates were monitored for 24 h, 48 h and 72 h.

**Results:**

Overall, 16 strains of *Acanthamoeba* isolates previously genotyped as T4 were cultivated axenically after several months. Thermotolerance and osmotolerance assay revealed that all of clinical strains, soil and animal feces strains were highly pathogenic isolates. Two dust and water strains did not grow at high temperature (42 °C) and osmolarity (1.5 M) and thus they were classified as weak pathogens.

**Conclusion:**

Most of T4 genotypes are highly pathogenic organisms. This is an important finding since *Acanthamoeba* belonging to T4 type is the predominate genotype in environmental and clinical samples. The presence of highly pathogenic *Acanthamoeba* may pose a risk within susceptible people.

## Introduction

*Acanthamoeba* are the ubiquities free-livingamebas with highly resistance to harsh conditions such as high osmolarity, extreme pH and high temperature (1). These amebas can be seen in two different forms including vegetative trophozoites and double walled cysts (2). To date, cellulose is amongst component of the cyst wall. Indeed, cellulose in the cyst wall introduces as a major component leading to resistance of cysts to stressful conditions (1). Of interest, *Acanthamoeba* were divided into 17 different genotypes (T1-T17) based on Diagnostic Fragment 3 of 18S rRNA gene (3). Although most of keratitis and amoebic granulomatousencephalitis isolates belong to T4 genotype, but there is not a defined pathogenicity correlation between a certain genotype and its pathogenic potential (4). Indeed, *Acanthamoeba* contains pathogenic strains especially within the T4 subgroup, but not all T4s are pathogenic despite the similarity of the SSU rDNA gene (4).

*Acanthamoeba* strains can show different pathogenic ability (5). Previous researches revealed that pathogenic *Acanthamoeba* can tolerate high temperature and osmolarity (5). This is an adaptation characteristic in these organisms, because pathogenic *Acanthamoeba* must cope with stressful situation of human body such as high osmolarity of tear film (4).

Based on the above characters Khan et al. have developed reliable and simple tests for pathogenic evaluation of *Acanthamoeba* strains. These tests were based on amoebae ability to survive in high osmolarity and temperature and they have been proved to discriminate between pathogenic and non-pathogenic strains (5, 6).

The main goal of the present study was to cultivate the *Acanthamoeba* in axenic culture and to assess the pathogenic ability of T4 genotypes from different clinical and environmental strains of *Acanthamoeba* using two physical assays. To the best of our knowledge, there were no previous surveys regarding pathogenic potential of *Acanthamoeba* strains using physical tests in Iran.

## Material and Methods

### Sources of strains

Sixteen *Acanthamoeba* strains including 10 environmental and 6 clinical strains were obtained during our previous research using culture method and PCR based assays (7–9). The sources of environmental strains were water [5], soil [2], dust [2] and cow feces [1]. These strains were genotyped by sequencing of highly variable Diagnostic Fragment 3 of the 18S rRNA gene and they were all genotyped as T4 type as previously described (7–9).

### Axenic cultivation of the isolates

In order to obtain an axenic culture, the amoebas of non-nutrient agar were cloned. Briefly, replicates were performed from the first line plates in order to eliminate bacterial and fungal contamination. These plates were then monitored daily. Approximately, after two months the clean plates containing only *Acanthamoeba* were achieved. Small piece of agar were then transferred to proteosepepton, yeast extract and glucose (PYG) medium (ATCC 712 PYG). This medium contained 0.4 M MgSO4, 7 H2O (10.0 ml), 0.05 M CaCl2 (8.0 ml), 0.1 M Sodium citrate, 2 H2O (34.0 ml), 0.005 M Fe (NH4)2(SO4)2, 6H2O (10.0 ml), 0.25 M Na2HPO4, 7 H2O (10.0 ml),0.25 M KH2PO4 (10.0 ml) and the pH was adjusted to 6.5 using KOH. Finally, the mentioned medium was supplemented with 2 M glucose (filter-sterilized) and was added aseptically to the solution (50.0 ml). Four isolates were cultivate axenic using TYI-S-33 medium which include glass-distilled water (100 ml), 0.1 g of potassium phosphate, dibasic; 0.06 g of potassium phosphate, monobasic; 0.2 g of sodium chloride; 0.2 g of casein digest peptone; 2 g of yeast extract; 1 g of glucose; 0.1 g of L-cysteine hydrochloride; 0.1 g of ascorbic acid; and 0.0023 g of ferric ammonium citrate (10). *Acanthamoeba* isolates were grown without shaking at room temperature. High amount of trophozoits were obtained within two days after successful axenic culture.

### Osmotolerance assay

Different osmolarityof non-nutrient agar plates were achieved using 0.5 M and 1 M mannitol (5). One hundred fifty µl of axenic culture in logarithmic phase (approximately 1000 trophozoites) were collected and were inoculated in 1% agar medium with different osmolarity and incubation were done at the room temperature. These plates were monitored for outgrowth of amoebae in 24 h, 48 h and 72 h.

### Thermotolerance assay

One hundred fifty µl of amoebae (approximately 1000 trophozoites) from axenic culture were divided into new culturemediums. Cultures were incubated at 37 °C and 42 °C for 24 h, 48 h and 72 h. Growth of *Acanthamoeba* spp. was evaluated by inverted microscopic examination up to 72 h. Besides, each *Acanthamoeba* strain was examined in triplicate in order to eliminate any probable error.

## Results

All of *Acanthamoeba* isolates previously genotyped as T4 were cultured axenically after several months. Indeed, the amoebas did not adapted to PYG medium readily. After adaptation of amoebas to PYG medium a large amount of trophozoites were achieved. Four isolate adapted to TYI-S-33 medium as mentioned above. Thermotolerance examination revealed that all of clinical isolates can grow at high temperatures including 37 °C and 42 °C. Soil and animal feces strains also were classified as highly pathogen by thermotolerance assays. It should be noted that two dust and five water strains have not grown at 42 °C and these isolates were classified as weakly pathogenic strains (Table 1).

**Table 1:** *Acanthamoeba* sources and their tolerance to different osmolarity and temperature

Code	Source	Osmolarity (Molar)		Temperature (°C)	
		0.5	1	37	42
CL1	Keratitis patients	+	+	+	+
CL2	Keratitis patients	+	+	+	+
CL3	Keratitis patients	+	+	+	+
CL4	Keratitis patients	+	+	+	+
CL5	Keratitis patients	+	+	+	+
CL6	Keratitis patients	+	+	+	+
W1	Water	+	−	+	−
W2	Water	+	−	+	−
W3	Water	+	−	+	−
W4	Water	+	−	+	−
W5	Water	+	−	+	−
F	Feces	+	+	+	+
S1	Soil	+	+	+	+
S2	Soil	+	+	+	+
D1	Dust	+	−	+	−
D2	Dust	+	−	+	−

Of interest, osmotolerance assay revealed that all clinical isolates have grown at high osmolarity (Table 1). Thus clinical strains were grouped within highly pathogenic *Acanthamoeba*. In accordance with thermotolerance assay, soil and animal strains also showed growth at high osmolarity. Dust and water isolates did not grow on high osmolarity. Based on thermotolerance and osmotolerance of these strains they were classified as weakly pathogenic *Acanthamoeba* (Table 1).

## Discussion

All of our isolates were cultivated axenically using two different culture mediums. The growth of four strains in TYI-S-33 medium is of interest and this medium could be an alternative culture medium for axenic cultivation of *Acanthamoeba* (9). Our study showed that most of T4 genotypes within our samples were classified as highly pathogenic strains, as they could grow in high temperature and high osmolarity. This is in agreement with previous studies (5). In fact, previous researches have shown that T4 genotype is the most commonly isolated genotypes from amoebic keratitis, granulomatous encephalitis and cutaneous acanthamoebiasis (1, 3, 7, 8). Genotyping based on 18S rRNA gene is not related to pathogenic ability. One of our interesting finding was that soil strains showed a high pathogenic ability. Therefore, environmental sources such as soil may be a risk factor for acquisition of amoebae infection. Additionally, highly pathogenic *Acanthamoeba* isolated from clinical samples could be an explanation for difficult treatment of *Acanthamoeba*-related infections.

Previous investigations showed that pathogenic *Acanthamoeba* must deal with different types of stresses (4, 11). This is the most important property that can lead to successful establishment of amoebae in human body. Indeed, highly virulent *Acanthamoeba* can withstand high osmolarity of tear film and thus it can lead to amoebic keratitis (4). One explanation of such property is the ability of pathogenic *Acanthamoeba* to secret high level of Heat Shock Proteins (HSP60 and HSP70). Based on aforementioned describe, Khan et al. set up a simple plating assay for identification of pathogenic *Acanthamoeba* from non-pathogenic strains (5). This plating test is mainly based on different tolerance of *Acanthamoeba* strains to variable osmolarity and temperature (4, 5). Previously, various methods such as extracellular proteases assay, use of animal models, cytopathic effects of isolates on cell culture and isoenzyme pattern were used for differentiation of pathogenic and non-pathogenic *Acanthamoeba*. These tests were time consuming and expensive (5). The advantages of plating assays are their easy performance and also they need minimal equipment in the laboratory.

## Conclusion

Most of T4 genotypes are highly pathogenic. This is an important finding since *Acanthamoeba* belonging to T4 type is the predominate genotype in environmental and clinical samples. The presence of highly pathogenic *Acanthamoeba* in environmental sources may pose a risk within susceptible people.
